# SMARCB1 Deficiency as a Driver of the Hallmarks of Cancer in Rhabdoid Tumours: Novel Insights into Dysregulated Energy Metabolism, Emerging Targets, and Ongoing Clinical Trials

**DOI:** 10.3390/metabo15050304

**Published:** 2025-05-03

**Authors:** Abdul L. Shakerdi, Graham P. Pidgeon

**Affiliations:** Department of Surgery, Trinity Translational Medicine Institute, St James’s Hospital & Trinity College Dublin, D08 NHY1 Dublin, Ireland

**Keywords:** rhabdoid tumours, SMARCB1 deficiency, hallmarks of cancer, energy metabolism, targeted therapy

## Abstract

**Background:** Rhabdoid tumours (RTs) are aggressive neoplasms most often characterised by biallelic loss of the SMARCB1 gene, encoding a core subunit of the SWI/SNF chromatin-remodelling complex. Despite their relative genetic stability, RTs exhibit a highly malignant phenotype and poor prognosis. **Methods:** This review explores the mechanisms underlying SMARCB1 aberrations, their role in driving hallmarks of cancer, and emerging therapeutic strategies for RTs. Ongoing clinical trials listed on ClinicalTrials were reviewed to evaluate the translational potential of targeted therapies in SMARCB1-deficient rhabdoid tumours. **Results:** Loss of SMARCB1 drives multiple cancer hallmarks by disrupting key regulatory pathways. It promotes unchecked cell proliferation through alterations in p16INK4a and Myc signalling. SMARCB1-deficient tumours possess immune-evading capabilities via PD-L1 overexpression and immune checkpoint activation. SMARCB1 deficiency also alters cellular energetics. The nucleotide biosynthesis pathway has been demonstrated to be upregulated in RT organoids, as shown by increased levels of pathway metabolites. Enzymes of the mevalonate pathway such as HMG-CoA reductase and mevalonate kinase are also dysregulated. Targeting glutathione metabolism with eprenetapopt may induce oxidative stress and apoptosis. Widespread epigenetic aberrations, including increased EZH2 activity, are being targeted with inhibitors such as tazemetostat. **Conclusions:** SMARCB1 loss is a central driver of cancer hallmarks in RTs, enabling proliferation, immune evasion, metabolic reprogramming, and epigenetic dysregulation. Future horizons in RT treatment include immunotherapies, epigenetic modifiers, and gene therapies. The synergy and optimal timing of targeted therapy with conventional treatment requires further characterisation for clinical translation.

## 1. Introduction

**Figure 1:**
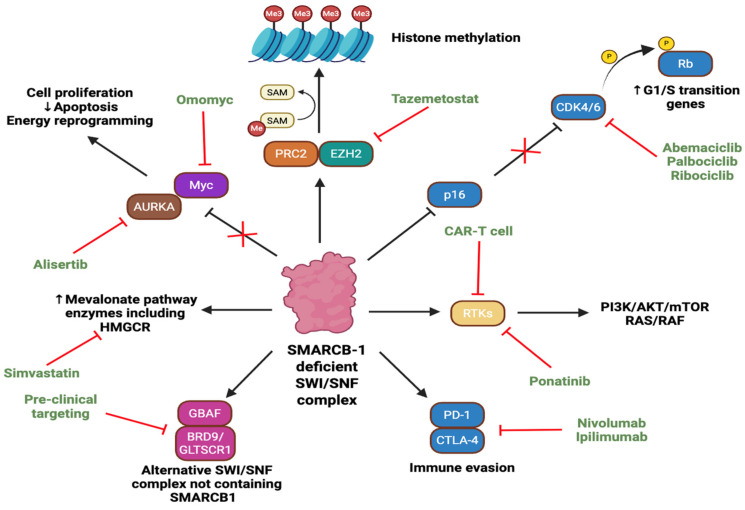
Aberrant pathways induced by SMARCB1 deficiency and corresponding therapeutic targets. ‘PRC2’ denotes polycomb repressive complex 2, ‘AURKA’ Aurora Kinase A, ‘RTK’ receptor tyrosine kinase, ‘BRD9’ bromodomain-containing protein 9, ‘GLTSCR1’ glioma tumour suppressor candidate region gene 1, ‘Rb’ retinoblastoma, ‘CDK4/6’ cyclin-dependent kinase 4/6, ‘PD-1’ programmed cell death protein 1, ‘CTLA-4’ cytotoxic T-lymphocyte–associated protein 4; SAM, S-adenosyl methionine, ‘PI3K’ phosphoinositide 3-kinase; ‘Akt’ protein kinase B; ‘mTOR’ mammalian target of rapamycin, and ‘HMGCR’ HMG-CoA reductase. Solid black arrows indicate activation or promotion of a protein or pathway. Solid bars with red cross indicate loss of inhibition. Red bars denote inhibition by corresponding drug or therapy. The symbol “P” in the yellow circle represents phosphorylation. Figure created with BioRender.com.

The SWI/SNF-related matrix-associated actin-dependent regulator of chromatin subfamily B member 1 (SMARCB1) gene is located on chromosome 22q11.2 and encodes a core subunit of the ATP-dependent SWI/SNF complex [1]. It is involved in chromatin remodelling and regulation of gene expression. Loss of the SMARCB1 gene has been linked to various neoplasms, including rhabdoid tumours (RTs), epithelioid sarcoma, and renal medullary carcinoma [2]. RTs are rare neoplasms which typically present in the paediatric population before the age of 3 [3]. They have been described in nearly all anatomical locations and are divided into those located intracranially, most commonly in the cerebellum [4], or extracranially in organs such as the kidney, gastrointestinal tract, and liver [5]. Apart from the frequent biallelic loss of SMARCB1, RTs are considered relatively genetically stable, despite their highly aggressive phenotype [6]. Germline mutations also predispose patients to various malignancies in a condition known as rhabdoid tumour predisposition syndrome [7]. Loss of SMARCB1 promotes cancer through various pathways (Figure 1), which will be described in more detail in this review. This article will also serve to discuss the mechanisms by which aberrations of the SMARCB1 gene occur and their implications in driving the various hallmarks of cancer and provide an overview of the novel therapeutic approaches in clinical trials for treatment of RTs.

The SMARCB1 gene extends over 50 kilobases, and its peptide sequence is encoded by nine exons [8]. The encoded protein has a molecular weight of 47 kDa, with a length of 385 amino acids. It is composed of four domains, including two highly conserved tandem repeat 1 and 2 domains (RPT1/2), flanked by the N-terminal Winged Helix DNA-binding domain and the C-terminal coiled-coil domain (CTD). Data derived from COSMIC v95 revealed 815 alterations in the SMARCB1 gene, largely clustered at the CTD. The majority of mutations (49.33%) were missense, followed by frameshift mutations (20.12%) and nonsense mutations (19.88%). Silent and inframe mutations accounted for approximately 10% of genetic aberrations [2].

Confirmation of the role of SMARCB1 as a tumour suppressor gene (TSG) arose when conditional gene knockout in adult mice resulted in CD8+ mature lymphoma and fully penetrant cancer formation [9]. Later studies were also able to produce RTs by inducing SMARCB1 deletion in earlier stages of embryonic development [10]. While complete loss of function of SMARCB1 is the best-characterised genetic mutation in RTs, some cases are associated with cytoplasmic localisation of the C-terminal truncated version of the gene, compromising its ability to exert its biological functions in the nucleus. Exportin 1 is thought to be responsible for this abnormal localisation through its interaction with the RPT2 domain of SMARCB1. As a result, nuclear export inhibitors such as leptomycin B and selinexor may be effective in these cases [11]. However, clinical efficacy is yet to be established [2].

## 2. Driver of the Hallmarks of Cancer

**Figure 2:**
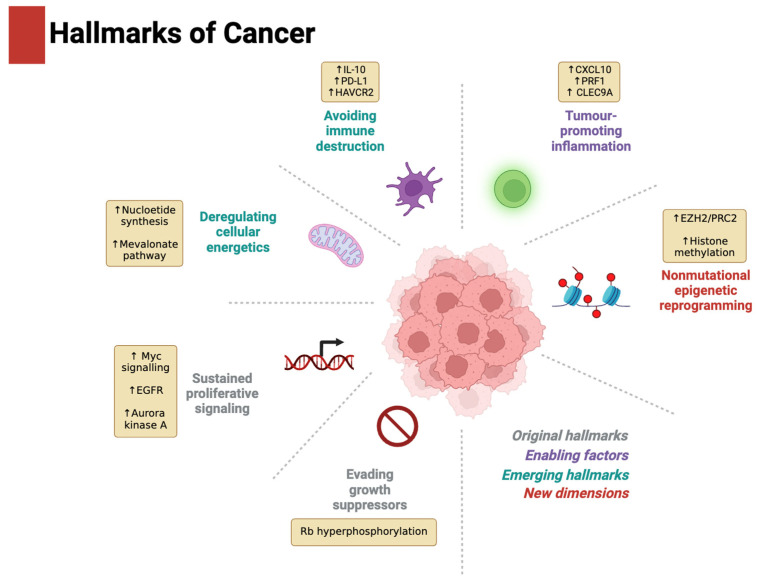
A summary of the role of SMARCB1 deficiency as a driver of the hallmarks of cancer in RTs. SMARCB1 loss promotes sustained proliferative signalling via upregulation of Myc, EGFR, and Aurora kinase A and facilitates evasion of growth suppressors through Rb hyperphosphorylation. Deregulated cellular energetics are marked by enhanced nucleotide synthesis and mevalonate pathway activation. It is important to note that upregulation of nucleotide synthesis has yet to be confirmed as a causal outcome of SMARCB1 deficiency. Tumour-promoting inflammation involves expression of CXCL10, PRF1, and CLEC9A, while immune evasion may be mediated through elevated IL-10, PD-L1, and HAVCR2. Non-mutational epigenetic reprogramming further supports oncogenesis and is driven by increased EZH2/PRC2 activity and histone methylation. Up arrows in figure indicate increased activity of protein or pathway. Figure created with BioRender.com.

The hallmarks of cancer were introduced by Hanahan and Weinberg in 2000 and were later expanded in 2011 and 2022 [12,13,14]. They describe a set of capabilities acquired by human cells during the carcinogenic process and provide a systematic structure for studying cancer, designing therapeutic targets, and guiding experimental models. Figure 2 summarises the role of SMARCB1 loss as a driver of the hallmarks of cancer in RTs.

### 2.1. Evading Growth Suppressors and Sustaining Proliferative Signalling

SMARCB1 has been shown to regulate the p16INK4a cyclin-dependent kinase (CDK) inhibitor which prevents the activation of the CDK4/6-cyclin D1 complex, responsible for Rb protein phosphorylation and hence the transcription of genes associated with G1/S phase progression [15]. SMARCB1 deficiency also enhances the activity of key oncogenic transcription factors, such as Myc, a helix–loop–helix protein that is one of the best-described drivers of cell proliferation [16]. Weissmiller and colleagues found that SNF5 encoded by SMARCB1 inhibits the DNA-binding ability of Myc, impeding its ability to recognise target genes. Further supporting these findings, the reintroduction of SNF5 into SMARCB1-null cells mimicked the primary transcriptional effects of MYC inhibition [17]. As a heterodimer with MAX, it regulates the expression of various genes involved in cell cycle regulation, apoptosis, angiogenesis, and cell metabolism [18,19,20]. OmoMYC (OMO-103) is a drug that targets Myc signalling by binding to c- and n-Myc, Max, and Miz1 and was shown to reduce RT cell viability in in vitro studies [17].

Another implicated oncogene is the epidermal growth factor receptor (EGFR). A phosphoproteomic study highlighted its abnormal expression in RTs and that its inhibition hinders cell proliferation [21]. MRTs have also been shown to exhibit coactivation of the receptor tyrosine kinases (RTKs) platelet-derived growth factor receptor A (PDGFRα) and fibroblast growth factor receptor 2 (FGFR-2) [22]. These are particularly attractive targets, as there exists a vast range of clinically approved RTK inhibitors. Dual targeting of these proteins individually or through the dual inhibitor ponatinib was shown to suppress the Akt and extracellular signal-regulated kinase (ERK1/2) pathways leading to apoptosis [22].

The use of small molecules aimed at recovering the lost function of TSGs in malignancy poses significant challenges, and many tumour suppressors are considered ‘undruggable’ for this reason [23]. Synthetic lethal relationships therefore provide a promising approach for the targeting of such genetic abnormalities [24]. Radko-Juettner et al., showed through a genome-wide CRISPR screen that the DDB1-CUL4-associated factor 5 (DCAF5) gene, which functions as a substrate receptor for E3-ubiquitin ligase, is required for the survival of SMARCB1-mutated RTs. In addition, depletion of DCAF5 allowed SWI/SNF to reaccumulate and restored histone marks such as H3K27ac and H3K4me1 at active enhancers, normalising cellular differentiation and reversing the cancer phenotype. These results suggest that SMARCB1 mutation leads to the destabilisation of the SWI/SNF complexes, which are subsequently cleared by the ubiquitin quality control pathway, in order to keep malignant cells viable [25]. Thus, targeting of DCAF5 may serve as a potential novel therapeutic approach in the treatment of RTs [26].

### 2.2. Tumour-Promoting Inflammation and Avoiding Immune Destruction

The microenvironment of certain subsets of RTs has been shown to be highly inflammatory and to express a high level of genes involved in CD8+ T cell activation, homing, and tumour infiltration, such as CXCL10, PRF1, and CLEC9A. Yet RTs deploy various survival mechanisms that allow them to thrive in such a hostile environment. Immunohistochemistry of RTs revealed overexpression of IL-10, programmed death ligand 1 (PD-L1), and PD-L1-expressing CD68+ myeloid cells, as well as other immune checkpoint proteins such as HAVCR2 [27]. One study found that in samples of 30 paediatric RTs, 47% stained positive for PD-L1 expression and therefore may have benefited from immune checkpoint inhibition [28]. However, several studies on atypical teratoid/rhabdoid tumours (ATRTs) have revealed the variable expression of PD-L1, ranging from 5.1% to 50%. This variability across studies may reflect differences in thresholds for positivity staining, the inclusion of partial staining, and the use of scoring systems such as the Cologne criteria [29]. Nevertheless, it remains no surprise that various clinical trials are currently examining the efficacy of a multitude of immune checkpoint blockers in the treatment of RTs, discussed in further detail later in this review.

### 2.3. Deregulating Cellular Energetics

**Figure 3:**
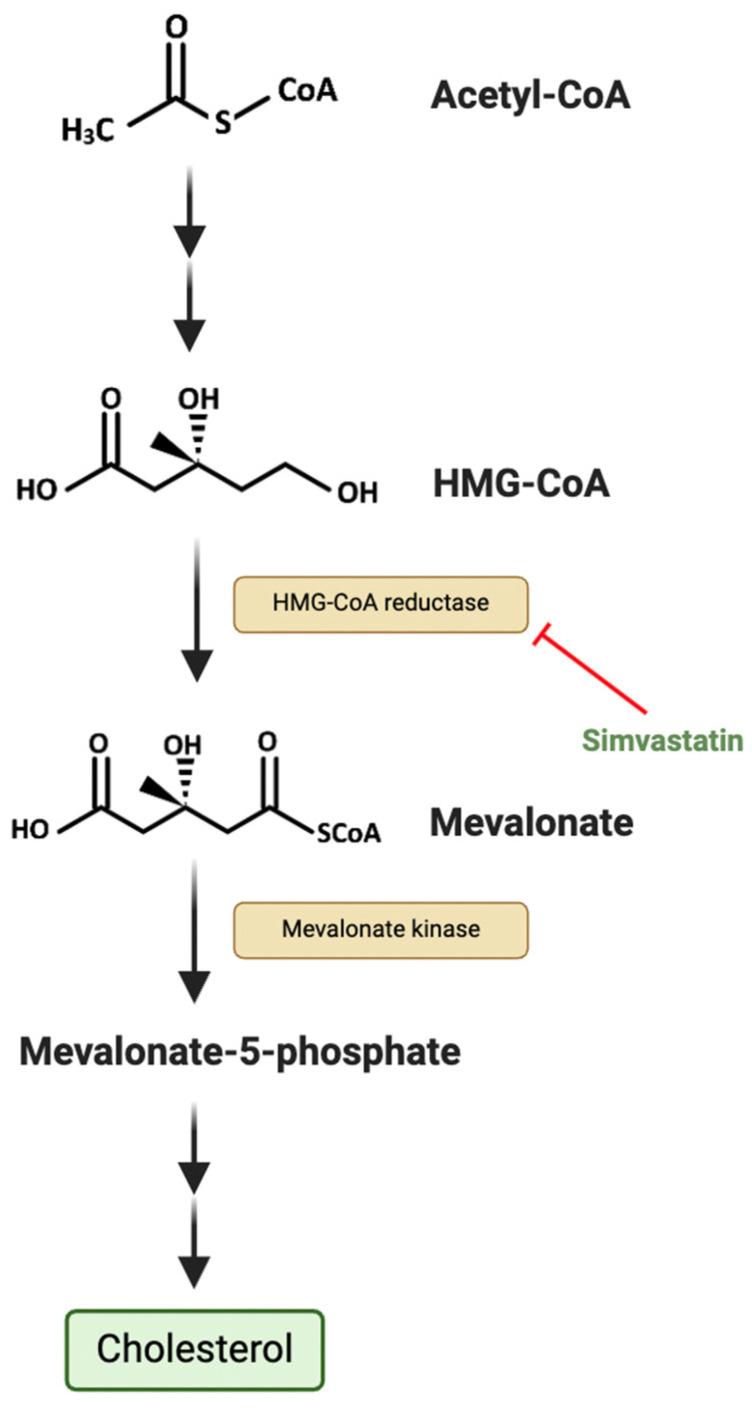
The mevalonate pathway, highlighting the inhibition of HMG-CoA reductase using simvastatin. ‘HMG-CoA’ denotes β-Hydroxy β-methylglutaryl-CoA. Figure created using BioRender.com.

An emerging metabolic target in SMARCB1-deficient tumours is the cholesterol biosynthetic pathway (Figure 3). Cholesterol plays a key role in the tumoural immune response by regulating the function of various immune cells, including dendritic cells, CD8⁺ T lymphocytes, NK cells, and γδ T cells [30]. One metabolite in the cholesterol biosynthetic pathway, isopentenyl pyrophosphate (IPP), regulates the activity of glutathione peroxidase 4 (GPX4), which inhibits ferroptosis to promote cancer cell survival [31]. In addition, cholesterol contributes to the maintenance of cancer stem cells by activating downstream of several developmental signalling pathways, including Hedgehog and Notch [32]. In a newly established RT cell line, it was found that restoration of SMARCB1 significantly downregulated the expression of enzymes such as HMG-CoA reductase (HMGCR) and mevalonate kinase and that the frequently prescribed cholesterol-lowering drug simvastatin induced apoptosis in RT cells [33]. A phase I study (NCT02390843) of simvastatin in combination with topotecan and cyclophosphamide in relapsed and/or refractory paediatric patients with solid and central nervous system (CNS) tumours, including RTs, has since been carried out and found that the combination was well tolerated [34]. It is important to note that cholesterol metabolism involves multiple highly regulated interconnected pathways [32]. Therefore, targeting a single enzyme or receptor within these pathways may not sufficiently inhibit tumour progression. In various solid tumours, including pancreatic cancer, statin treatment has been demonstrated to induce a compensatory increase in HMGCR and exert a partial epithelial-to-mesenchymal transition (EMT) phenotype [35]. EMT is a key process in oncogenesis which confers migratory and metastatic properties to malignant epithelial cells [36]. A potential advantage in the clinical translation of statins in RTs is that they have been increasingly prescribed in paediatric populations with conditions like familial hypercholesterolemia, meaning that their safety and dosing parameters are relatively well defined [37].

**Figure 4:**
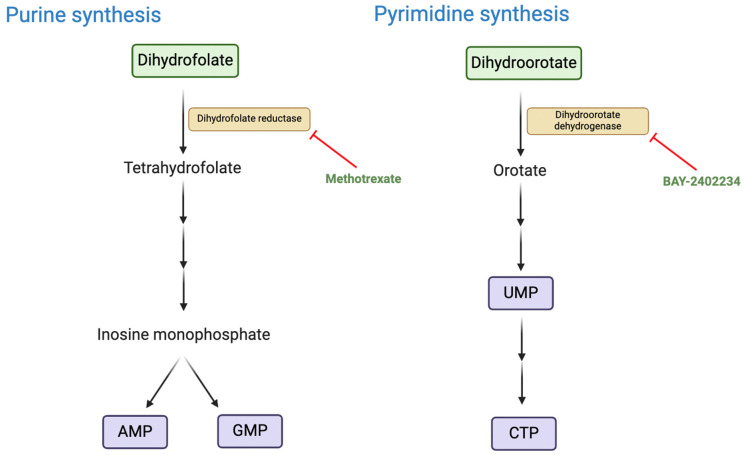
The nucleotide biosynthetic pathway as a potential metabolic vulnerability in RTs. ‘AMP’ denotes adenosine monophosphate, ‘GMP’ guanine monophosphate, ‘UMP’ uridine monophosphate, and ‘CTP’ cytidine triphosphate. Figure created using BioRender.com.

A recent study by Kes and colleagues used patient-derived organoids to investigate the metabolic vulnerabilities of RTs [38]. Gene expression analysis and liquid chromatography–mass spectrometry revealed that de novo nucleotide biosynthesis is significantly upregulated in RTs, as demonstrated by the increased levels of metabolites such as uridine monophosphate (UMP) and inosine monophosphate (IMP). Pharmacological inhibition (Figure 4) using methotrexate and BAY-2402234, targeting purine and pyrimidine synthesis pathways, respectively, resulted in significant cytotoxic effects in RT tumoroids while sparing normal kidney organoids. Further in vivo validation in RT xenograft mouse models demonstrated that methotrexate treatment delayed tumour progression [38]. While the direct link between these metabolic aberrations and SMARCB1 loss has not been confirmed, SMARCB1 loss is likely to be indirectly or directly causal given that it is the defining driver of RTs. Further research into the precise role of SMARCB1 deficiency as a driver of nucleotide synthesis may yield novel therapeutic targets and enhance understanding of the pathogenesis of the disease.

### 2.4. Non-Mutational Epigenetic Reprogramming

Widespread transcriptional dysregulation and aberrant chromatin remodelling is known to occur as a result of SMARCB1 loss in RTs [39]. In mouse embryonic fibroblasts, SMARCB1 deletion resulted in decreased levels of the active histone markers H3K27ac and H3Kme1 at enhancers [40]. The action of the polycomb repressive complex 2 (PRC2), whose catalytic subunit EZH2 exhibits histone methyltransferase activity, is opposed by the SWI/SNF complex. As a result, SMARCB1 mutation is associated with uncontrolled PRC2-mediated inhibition of BRG1/BRM-associated factor (BAF) target genes. Tazemetostat, an inhibitor of EZH2, has received FDA approval for treatment of SMARCB1-deficient epithelioid sarcoma [41], and trials for other malignancies, including RTs, are ongoing. In the absence of SMARCB1, RT cells upregulate GLTSCR1-like-containing BAF complex (GBAF) expression, an SWI/SNF subcomplex which does not contain the SMARCB1 subunit. GBAF’s subunits BRD9 and GLTSCR1 are proposed drug targets, and their degradation or inhibition has been shown to reduce the viability of RT cell lines [42]. SMARCB1 deficiency also prevents the recruitment of cBAF and PBAF to the promoter and enhancer regions of SLC7A11, which plays a crucial role in glutathione metabolism by supplying cysteine for glutathione synthesis. Treatment with eprenetapopt has been shown to further decrease glutathione levels, resulting in oxidative stress and apoptosis [43].

**Figure 5:**
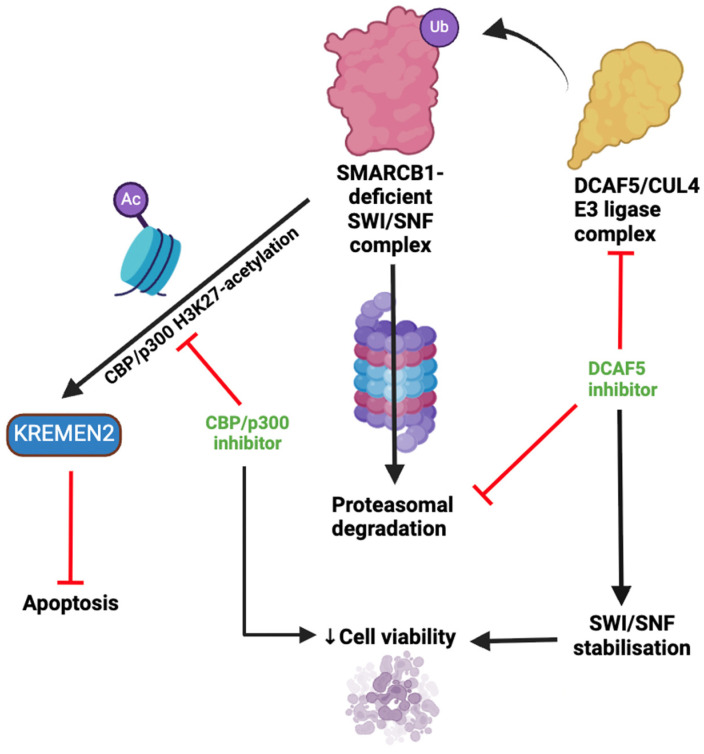
Synthetic lethal vulnerabilities in RTs. In SMARCB1-deficient cells, SMARCB1-deficient SWI/SNF complexes are targeted for ubiquitination and proteasomal degradation by the DCAF5/CUL4 E3 ligase complex. Inhibition of DCAF5 stabilises these defective SWI/SNF complexes, leading to toxic accumulation and loss of cell viability. In addition, CBP/p300-mediated H3K27 acetylation upregulates KREMEN2 expression, which suppresses apoptosis and promotes cell survival. Inhibition of CBP/p300 reduces KREMEN2 levels, promoting apoptosis. ‘Ub’ in purple circle indicates ubiquitination, while ‘Ac’ indicates acetylation. Figure created with BioRender.com.

It has recently been demonstrated that in SMARCB1-deficient cells, CBP/p300-mediated H3K27 acetylation promotes the expression of Kringle-containing transmembrane protein 2 (*KREMEN2)* [44], encoding a protein which complexes with KREMEN1 to regulate the Wnt/β catenin pathway [45] and inhibit apoptosis [46]. SMARCB1-deficient cells subsequently become dependent on KREMEN2 for survival. Dual inhibitors of CBP/p300 have been shown to suppress growth and induce apoptosis in cell lines and xenograft models of SMARCB1-deficient cells but not SMARCB1-expressing cells [44]. This serves as another example of synthetic lethal vulnerabilities in SMARCB-1-deficient RTs, which are highlighted in Figure 5.

### 2.5. Activating Invasion and Metastasis

While the link between SMARCB1 deficiency and enhanced metastatic potential has been explored in several tumour types, this association has been somewhat understudied in RTs. A recent study in bladder cancer showed that SMARCB1-deficient tumours displayed increased IL6–janus kinase–signal transducer and activator of transcription 3 (IL6-JAK-STAT3) signalling in in vivo models and patient tumours, believed to be associated with metastasis. STAT3 knockdown reduced tumour growth and lung metastasis. In addition, treatment of xenografts with TTI-101, a selective inhibitor of phosphorylated STAT3, reduced metastatic burden as measured by bioluminescence imaging [47]. STAT3 has been shown to promote the expression of Twist and Snail, master regulators of EMT [48]. In addition, a study by Liu and colleagues [49] found that cisplatin-resistant ATRT cells exhibit enhanced invasion, motility, and stem-like properties driven by upregulation of the STAT3–Snail axis. Knockdown of STAT3 was shown to suppress Snail expression, reduce invasiveness, and reverse resistance, while STAT3 inhibition improved survival in ATRT-CisR-transplanted mice. This highlights how STAT3 expression is not only involved in promoting an aggressive disease phenotype but also may be implicated in therapeutic failure. In a case report of poorly differentiated sacrococcygeal chordoma, expression of the premetastatic chemokine receptor CXCR4 was upregulated [50]. This may be explainable by the upregulation of EZH2 in SMARCB1-deficient tumours, which enhances CXCR4 expression through the repression of the tumour-suppressive microRNAs miR-622 and miR-9 [51,52]. However, it is crucial to note that the patient’s tumour also displayed TP53 mutation and RB1 loss, so these premetastatic alterations cannot be attributed with certainty to SMARCB1 loss, particularly in a single patient report.

## 3. Ongoing Trials and Future Directions

### 3.1. Immunotherapies

Immune checkpoint proteins (ICPs) are molecules which control the activation of T lymphocytes, a mechanism employed by the body in order to limit the indiscriminate killing of healthy tissues [53]. Various ICPs have been discovered to date, the most notable being PD-1 and cytotoxic T-lymphocyte-associated protein 4 (CTLA-4). PD-1 on T cells binds to PD-L1 expressed on tumour cells and antigen-presenting cells to suppress T cell proliferation, activity, and cytokine production [54]. CTLA-4 competes with CD28 for the binding of co-stimulatory molecules CD80/86 [55]. A more recently discovered ICP is T cell immunoreceptor with Ig and ITIM domains (TIGIT), which inhibits T and NK cell activation through interaction with CD155 [56].

Immune checkpoint inhibitors (ICIs) have been hailed as exciting anti-cancer therapeutics in the last few decades. Single-agent inhibition of PD-1 or PD-L1 in adults has demonstrated promising activity in various solid tumours. However, in the paediatric population, efficacy with mono-blockade has been limited to just a few tumours, particularly lymphomas [57]. Various ongoing clinical trials are therefore investigating the efficacy of combinations of ICIs in the treatment of SMARCB-1-deficient RTs. A phase II trial (NCT04416568) is currently recruiting patients to investigate the combination of nivolumab (anti-PD1) with ipilimumab (anti-CTLA4) in children and young adults with SMARCB1-deficient tumours, including RTs, epithelioid sarcomas, and chordomas [58]. In mouse models of solid tumours, co-blockade of both PD-L1 and TIGIT has been shown to promote the clonal expansion of multipotent anti-tumoural T lymphocytes [59]. Inhibition of PD-L1 and TIGIT through atezolizumab and tiragolumab, respectively, is being examined in a phase I/II clinical trial for the treatment of relapsed or refractory SMARCB1- or SMARCA4-deficient tumours [60]. Another proposed mechanism of enhancing ICI efficacy is through simultaneous inhibition of EZH2, a histone methyltransferase which has been shown to suppress MHC-I presentation and upregulate PD-L1 [61]. TAZNI (NCT05407441) is a phase I/II study recruiting to evaluate the combination of an EZH2 inhibitor, tazemetostat, with nivolumab and ipilimumab for SMARCA4-/SMARCB-1-deficient paediatric malignancies [62]. Table 1 summarises the ongoing clinical trials utilising ICIs in RTs. 

Chimeric antigen receptor T (CAR-T) cells are genetically engineered T lymphocytes that express synthetic receptors designed to specifically recognise and bind target antigens on tumour cells [63]. This results in the activation of intracellular signalling domains such as CD3ζ, 4-1BB, and CD28 to elicit cancer cytotoxicity [64]. Multiple CAR-T cell strategies are being investigated for RTs (Table 2). CAR-T cells are often targeted against over-expressed tumour antigens, such as the membrane-bound proteoglycan glypican 3 (GPC3), in the case of solid tumours, including RTs. These trials (NCT05103631, NCT04715191, and NCT04377932) are investigating the use of IL-15/21 to enhance CAR-T cell efficacy and persistence [65,66,67]. CAR-T cells may also serve as ICIs. For example, a couple of trials (NCT05835687 and NCT04897321) are exploring the targeting of B7-H3, a transmembrane immunoregulatory protein, by CAR-T cells for the treatment of solid tumours.

**Table 1:** Active clinical trials utilising immune checkpoint inhibitors to target RTs.

Trial Number	Phase	SMARCB-1 Targeting Therapies	Disorder	Target	Status	Sites	Primary Outcome Measures
NCT04416568 [58]	II	Nivolumab and Ipilimumab	SMARCB1-negative tumours	PD-1, CTLA-4	Recruiting	Texas, USA	Objective overall response rate
NCT05286801 [60]	I/II	Tiragolumab and Atezolizumab	SMARCB1- or SMARCA4-deficient tumours, including RTs	TIGIT, PD-L1	Recruiting	USA, Canada, and Australia	Objective response rate and dose-limiting toxicities
NCT05407441 [62]	I/II	Tazemetostat, nivolumab, ipilimumab	SMARCB1-negative or SMARCA4-deficient tumours, including RTs	EZH2, PD-1, CTLA-4	Recruiting	Boston, MA, USA	Toxicity and dosing parameters
NCT06622941 [68]	II	Nivolumab (ONO-4538)	RTs	PD-1	Not yet recruiting	Osaka and Tokyo, Japan	Objective response rate

**Table 2:** Active clinical trials utilising CAR-T cells and cytotoxic lymphocytes against RTs.

Trial Number	Phase	SMARCB-1-Targeting Therapies	Disorder	Target	Status	Sites	Primary Outcome Measures
NCT06193759 [69]	I	Cytotoxic T lymphocytes directed against proteogenomically determined tumour-specific antigens	Paediatric brain tumours, including RTs	Various tumour-specific antigens	Recruiting	Washington, USA	Various adverse events and toxicity parameters
NCT05835687 [70]	I	Locoregional autologous B7-H3-CAR T cells	Primary CNS neoplasms, including RTs	B7-H3-positive tumours	Recruiting	Tennessee, USA	Maximum tolerated dose
NCT05103631 [65]	I	GPC3-CART cells and IL-15	Solid tumours, including RTs	GPC3-positive tumours	Recruiting	Texas, USA	Dose-limiting toxicities
NCT04897321 [71]	I	Autologous B7-H3-CAR T cells	Solid tumours, including RTs	B7-H3-positive tumours	Recruiting	Tennessee, USA	Maximum tolerated dose
NCT04715191 [66]	I	GPC3-CART cells and IL-15/21	Paediatric solid tumours, including RTs	GPC3-positive tumours	Recruiting	Texas, USA	Dose-limiting toxicities
NCT04377932 [67]	I	GPC3-CART cells and IL-15	Paediatric solid tumours, including RTs	GPC3-positive tumours	Recruiting	Texas, USA	Dose-limiting toxicities
NCT04185038 [72]	I	Locoregional autologous B7-H3-CAR T cells	Paediatric CNS tumours, including RTs	B7-H3-positive tumours	Recruiting	Washington, USA	Feasibility and adverse event parameters
NCT03618381 [73]	I	EGFR806 CAR T cell immunotherapy	Recurrent/refractory solid tumours in children and young adults, including RTs	EGFR	Recruiting	Washington, USA	Maximum tolerated dose, feasibility, and adverse event parameters
NCT04483778 [74]	I	B7-H3-CAR T cells	Recurrent/refractory solid tumours in children and young adults, including RTs	B7-H3-positive tumours	Active, not recruiting	Washington, USA	Various safety, tolerability, toxicity, and feasibility parameters

### 3.2. Targeting Epigenetic Aberrations

The role of epigenetics in cancer development and progression has been spotlighted as a key area of interest in recent years. In 2024, an article by Esteller and colleagues proposed the concept of six potentially targetable epigenetic hallmarks of cancer [75]. Epigenetic therapies in cancer aim to modify aberrant epigenetic patterns, such as DNA methylation, histone modifications, and chromatin remodelling [76]. The phase I study of tazemetostat, which targets the PRC2 catalytic subunit EZH2 (NCT02601937), included paediatric patients with relapsed or refractory RTs, other SMARCB1-deficient tumours, and synovial sarcoma [77]. Objective responses were seen in 14% of the dose-expansion cohort population, with a 24% response in intracerebral RT patients [78].

Vorinostat is an agent which inhibits the activity of histone deacetylases [79]. A phase I trial (NCT01076530) investigated the combination of vorinostat with temozolomide for the treatment of relapsed/refractory paediatric CNS tumours [80]. Nineteen patients were recruited, including two with rhabdoid tumours. Clinical efficacy was modest, with only three patients exhibiting stable disease and one having a partial response. Nevertheless, the combination was well tolerated, and myelosuppression was identified as the dose-limiting toxicity. It was also noted that accumulation of acetylated H3 histone in peripheral blood mononuclear cells occurred following administration of vorinostat [81]. While clinical utility with epigenetic targeting appears limited thus far, it remains a relatively novel field of cancer research. Future studies focused on identifying predictive biomarkers and exploring synergistic combinations may improve outcomes.

### 3.3. Targeting Auxiliary Oncogenic Aberrations

Aurora kinase A (AURKA) plays a pivotal role in mitotic spindle formation, and its overexpression has been implicated in various malignancies, including RTs [82,83]. A phase II clinical trial evaluated alisertib, an AURKA inhibitor, as a monotherapy in patients under 22 years with recurrent or progressive ATRTs. The study did not meet its primary efficacy endpoint of ≥10 patients being progression-free at 12 weeks. However, alisertib was found to be well tolerated, with a 6-month progression-free survival (PFS) of 31% ± 8.2%. A third of patients also demonstrated disease stabilisation beyond 6 months [84]. Mutations leading to truncated SMARCB1 can result in its exportin-1-mediated mislocalisation in the cytoplasm and hence loss of tumour-suppressive capacity. A phase I trial of the exportin-1 inhibitor selinexor for paediatric patients with recurrent/refractory solid and CNS tumours established the maximal tolerated dose and recommended a phase II (NCT05985161) starting dose of 35 mg/m^2^ for patients with recurrent/progressive ATRTs [85]. The proteins CDK4/6 are crucial for cell cycle progression. A phase I trial of the CDK4/6 inhibitor ribociclib in paediatric patients with RTs, neuroblastoma, and other solid tumours showed acceptable safety and prolonged stable disease in a subset of patients [86]. A phase II study is currently recruiting to assess the efficacy of ribociclib with topotecan and temozolomide in paediatric patients with relapsed or refractory neuroblastoma and solid tumours, including RTs [87]. Table 3 provides an overview of the active clinical trials targeting aberrations in the cell cycle (CDK4/6) and the dysregulated nuclear export of SMARCB1 (exportin-1). Future efforts should attempt to identify predictive biomarkers to stratify patients most likely to benefit from inhibitors of these auxiliary oncogenic aberrations.

### 3.4. Next Frontiers in Rhabdoid Tumour Research and Treatment

Gene therapy holds promise as a potential approach to treating cancers through the modification or restoration of gene expression [90]. A study published by Kim et al. in 2024 explored the use of scL-SMARCB1, a nanomedicine used to deliver wild-type SMARCB1 into ATRT cells via transferrin receptor-mediated endocytosis [91]. scL-SMARCB1 therapy was found to upregulate the expression of MYC-repressed genes such as CDKN1C and CDKN2A, while downregulating MYC-activated genes. AURKA expression was also significantly suppressed. It was also found that transfection with SMARCB1 potentiated cytotoxicity induced by cisplatin and radiation therapy [91]. This highlights how genetic therapy may not only restore biological markers in rhabdoid tumours but also enhance their sensitivity to conventional chemoradiation. This may have the potential to increase therapeutic efficacy and limit toxicity to healthy tissue.

Artificial intelligence (AI) may serve as a powerful tool for analysing complex genomic data in order to identify new therapeutic indications for drugs. One study has described the application of the Response Algorithm for Drug Positioning and Rescue (RADR^®^) AI platform, combined with the CellMiner Cross Database, in uncovering novel therapeutic insights for the acylfulvene-derivative anti-cancer drugs LP-100 and LP-184 [92]. Gene set enrichment analysis (GSEA) revealed a significant negative correlation between LP-184 sensitivity and SMARCB1 expression. These computational findings were then validated in SMARCB1-deficient ATRT cell lines and through in vivo experiments [92]. This demonstrates the utility of AI-driven platforms in rapidly identifying novel therapeutic indications for drugs. Such an approach may be especially useful for rarer cancers such as SMARCB1-deficient rhabdoid malignancies, which tend to have a relatively limited specific research focus.

## 4. Conclusions

The discussed trials and future innovations highlight how personalised therapies based on genomic, metabolomic, and immunological profiling have the potential to be the mainstay of RT treatment and disease monitoring. Emerging evidence also highlights the importance of metabolic reprogramming in RTs, with pathways such as cholesterol biosynthesis and nucleotide metabolism presenting novel therapeutic vulnerabilities. Integrating metabolomic profiling into future studies may uncover additional biomarkers and enable the development of metabolism-targeted therapies in SMARCB1-deficient tumours. Identifying subsets of patients who are most likely to respond to the various treatments will be crucial. Pre-clinical models such as xenografts may not fully recapitulate the RT microenvironment or the dynamics of tumour progression. Thus, systems such as patient-derived organoid systems may offer promising avenues to better mimic in vivo conditions and improve translational relevance. The effects, optimal timing, and integration of combinations of SMARCB1-targeted treatments with conventional therapies must be better characterised in order to enhance patient outcomes and allow incorporation into later-phase clinical trials and clinical practice. Additional research and genomic analysis are required to further elucidate the role of SMARCB1 in tumour suppression and to identify additional molecular targets.

## Figures and Tables

**Table 3:** Clinical trials targeting the cell cycle checkpoint control, the dysregulated nuclear export of SMARCB1, and Aurora kinase A.

Trial Number	Phase	SMARCB-1-Targeting Therapies	Disorder	Target	Status	Sites	Primary Outcome Measures
NCT05429502 [87]	I/II	Ribociclib	Solid tumours, including RTs	CDK4/6	Recruiting	USA, UK, Spain, Singapore, Italy, Germany, France, and Australia	Overall response rate and dose-limiting toxicities
NCT05985161 [88]	II	Selinexor	Solid tumours, including RTs	Exportin-1	Recruiting	Various USA sites	Complete and partial response
NCT03709680 [89]	I/II	Palbociclib	Recurrent/refractory solid tumours, including RTs	CDK4/6	Active, not recruiting	>10 countries, including USA, UK, Brazil, and Korea	Event-free survival, first-cycle dose-limiting toxicities, frequency of adverse events, complete response or partial response

## Data Availability

No new data has been created outside of this manuscript.

## References

[1] Pawel B.R. (2017). SMARCB1-deficient Tumors of Childhood: A Practical Guide. Pediatr. Dev. Pathol..

[2] Cooper G.W., Hong A.L. (2022). SMARCB1-Deficient Cancers: Novel Molecular Insights and Therapeutic Vulnerabilities. Cancers.

[3] Gupta N.K., Godbole N., Sanmugananthan P., Gunda S., Kasula V., Baggett M., Gajjar A., Kouam R.W., D’Amico R., Rodgers S. (2024). Management of Atypical Teratoid/Rhabdoid Tumors in the Pediatric Population: A Systematic Review and Meta-Analysis. World Neurosurg..

[4] Nemes K., Bens S., Bourdeaut F., Johann P., Kordes U., Siebert R., Frühwald M.C., Adam M.P., Feldman J., Mirzaa G.M., Pagon R.A., Wallace S.E., Amemiya A. (1993). Rhabdoid Tumor Predisposition Syndrome. GeneReviews^®^.

[5] Sultan I., Qaddoumi I., Rodríguez-Galindo C., Nassan A.A., Ghandour K., Al-Hussaini M. (2010). Age, stage, and radiotherapy, but not primary tumor site, affects the outcome of patients with malignant rhabdoid tumors. Pediatr. Blood Cancer.

[6] Lee R.S., Stewart C., Carter S.L., Ambrogio L., Cibulskis K., Sougnez C., Lawrence M.S., Auclair D., Mora J., Golub T.R. (2012). A remarkably simple genome underlies highly malignant pediatric rhabdoid cancers. J. Clin. Investig..

[7] Nemes K., Fruhwald M.C. (2018). Emerging therapeutic targets for the treatment of malignant rhabdoid tumors. Expert Opin. Ther. Targets.

[8] Kalimuthu S.N., Chetty R. (2016). Gene of the month: SMARCB1. J. Clin. Pathol..

[9] Roberts C.W., Leroux M.M., Fleming M.D., Orkin S.H. (2002). Highly penetrant, rapid tumorigenesis through conditional inversion of the tumor suppressor gene Snf5. Cancer Cell.

[10] Han Z.Y., Richer W., Freneaux P., Chauvin C., Lucchesi C., Guillemot D., Grison C., Lequin D., Pierron G., Masliah-Planchon J. (2016). The occurrence of intracranial rhabdoid tumours in mice depends on temporal control of Smarcb1 inactivation. Nat. Commun..

[11] Pathak R., Zin F., Thomas C., Bens S., Gayden T., Karamchandani J., Dudley R.W., Nemes K., Johann P.D., Oyen F. (2021). Inhibition of nuclear export restores nuclear localization and residual tumor suppressor function of truncated SMARCB1/INI1 protein in a molecular subset of atypical teratoid/rhabdoid tumors. Acta Neuropathol..

[12] Hanahan D., Weinberg R.A. (2011). Hallmarks of cancer: The next generation. Cell.

[13] Hanahan D. (2022). Hallmarks of Cancer: New Dimensions. Cancer Discov..

[14] Hanahan D., Weinberg R.A. (2000). The hallmarks of cancer. Cell.

[15] Betz B.L., Strobeck M.W., Reisman D.N., Knudsen E.S., Weissman B.E. (2002). Re-expression of hSNF5/INI1/BAF47 in pediatric tumor cells leads to G1 arrest associated with induction of p16ink4a and activation of RB. Oncogene.

[16] García-Gutiérrez L., Delgado M.D., León J. (2019). MYC Oncogene Contributions to Release of Cell Cycle Brakes. Genes.

[17] Weissmiller A.M., Wang J., Lorey S.L., Howard G.C., Martinez E., Liu Q., Tansey W.P. (2019). Inhibition of MYC by the SMARCB1 tumor suppressor. Nat. Commun..

[18] Amati B., Land H. (1994). Myc—Max—Mad: A transcription factor network controlling cell cycle progression, differentiation and death. Curr. Opin. Genet. Dev..

[19] Baudino T.A., McKay C., Pendeville-Samain H., Nilsson J.A., Maclean K.H., White E.L., Davis A.C., Ihle J.N., Cleveland J.L. (2002). c-Myc is essential for vasculogenesis and angiogenesis during development and tumor progression. Genes Dev..

[20] Dong Y., Tu R., Liu H., Qing G. (2020). Regulation of cancer cell metabolism: Oncogenic MYC in the driver’s seat. Signal Transduct. Target. Ther..

[21] Darr J., Klochendler A., Isaac S., Geiger T., Eden A. (2015). Phosphoproteomic analysis reveals Smarcb1 dependent EGFR signaling in Malignant Rhabdoid tumor cells. Mol. Cancer.

[22] Wong J.P., Todd J.R., Finetti M.A., McCarthy F., Broncel M., Vyse S., Luczynski M.T., Crosier S., Ryall K.A., Holmes K. (2016). Dual Targeting of PDGFRα and FGFR1 Displays Synergistic Efficacy in Malignant Rhabdoid Tumors. Cell Rep..

[23] Gregory G.L., Copple I.M. (2023). Modulating the expression of tumor suppressor genes using activating oligonucleotide technologies as a therapeutic approach in cancer. Mol. Ther. Nucleic Acids.

[24] Ge M., Luo J., Wu Y., Shen G., Kuang X. (2024). The biological essence of synthetic lethality: Bringing new opportunities for cancer therapy. MedComm—Oncology.

[25] Radko-Juettner S., Yue H., Myers J.A., Carter R.D., Robertson A.N., Mittal P., Zhu Z., Hansen B.S., Donovan K.A., Hunkeler M. (2024). Targeting DCAF5 suppresses SMARCB1-mutant cancer by stabilizing SWI/SNF. Nature.

[26] Dedes K.J., Wilkerson P.M., Wetterskog D., Weigelt B., Ashworth A., Reis-Filho J.S. (2011). Synthetic lethality of PARP inhibition in cancers lacking BRCA1 and BRCA2 mutations. Cell Cycle.

[27] Chun H.E., Johann P.D., Milne K., Zapatka M., Buellesbach A., Ishaque N., Iskar M., Erkek S., Wei L., Tessier-Cloutier B. (2019). Identification and Analyses of Extra-Cranial and Cranial Rhabdoid Tumor Molecular Subgroups Reveal Tumors with Cytotoxic T Cell Infiltration. Cell Rep..

[28] Forrest S.J., Al-Ibraheemi A., Doan D., Ward A., Clinton C.M., Putra J., Pinches R.S., Kadoch C., Chi S.N., DuBois S.G. (2020). Genomic and Immunologic Characterization of INI1-Deficient Pediatric Cancers. Clin. Cancer Res..

[29] Tran S., Plant-Fox A.S., Chi S.N., Narendran A. (2023). Current advances in immunotherapy for atypical teratoid rhabdoid tumor (ATRT). Neurooncol. Pract..

[30] Pecci F., Cognigni V., Giudice G.C., Paoloni F., Cantini L., Saini K.S., Abushukair H.M., Naqash A.R., Cortellini A., Mazzaschi G. (2025). Unraveling the link between cholesterol and immune system in cancer: From biological mechanistic insights to clinical evidence. A narrative review. Crit. Rev. Oncol./Hematol..

[31] Ye L., Wen X., Qin J., Zhang X., Wang Y., Wang Z., Zhou T., Di Y., He W. (2024). Metabolism-regulated ferroptosis in cancer progression and therapy. Cell Death Dis..

[32] Xiao M., Xu J., Wang W., Zhang B., Liu J., Li J., Xu H., Zhao Y., Yu X., Shi S. (2023). Functional significance of cholesterol metabolism in cancer: From threat to treatment. Exp. Mol. Med..

[33] Matsumoto F., Yokogami K., Yamada A., Moritake H., Watanabe T., Yamashita S., Sato Y., Takeshima H. (2024). Targeting cholesterol biosynthesis for AT/RT: Comprehensive expression analysis and validation in newly established AT/RT cell line. Hum. Cell.

[34] Cash T., Jonus H.C., Tsvetkova M., Beumer J.H., Sadanand A., Lee J.Y., Henry C.J., Aguilera D., Harvey R.D., Goldsmith K.C. (2023). A phase 1 study of simvastatin in combination with topotecan and cyclophosphamide in pediatric patients with relapsed and/or refractory solid and CNS tumors. Pediatr Blood Cancer.

[35] Dorsch M., Kowalczyk M., Planque M., Heilmann G., Urban S., Dujardin P., Forster J., Ueffing K., Nothdurft S., Oeck S. (2021). Statins affect cancer cell plasticity with distinct consequences for tumor progression and metastasis. Cell Rep..

[36] Ribatti D., Tamma R., Annese T. (2020). Epithelial-Mesenchymal Transition in Cancer: A Historical Overview. Transl. Oncol..

[37] Fiorentino R., Chiarelli F. (2023). Statins in Children, an Update. Int. J. Mol. Sci..

[38] Kes M.M.G., Morales-Rodriguez F., Zaal E.A., de Souza T., Proost N., van de Ven M., van den Heuvel-Eibrink M.M., Jansen J.W.A., Berkers C.R., Drost J. (2025). Metabolic profiling of patient-derived organoids reveals nucleotide synthesis as a metabolic vulnerability in malignant rhabdoid tumors. Cell Rep. Med..

[39] Kenny C., O’Meara E., Ulas M., Hokamp K., O’Sullivan M.J. (2021). Global Chromatin Changes Resulting from Single-Gene Inactivation-The Role of SMARCB1 in Malignant Rhabdoid Tumor. Cancers.

[40] Wang X., Lee R.S., Alver B.H., Haswell J.R., Wang S., Mieczkowski J., Drier Y., Gillespie S.M., Archer T.C., Wu J.N. (2017). SMARCB1-mediated SWI/SNF complex function is essential for enhancer regulation. Nat. Genet..

[41] Hoy S.M. (2020). Tazemetostat: First Approval. Drugs.

[42] Wang X., Wang S., Troisi E.C., Howard T.P., Haswell J.R., Wolf B.K., Hawk W.H., Ramos P., Oberlick E.M., Tzvetkov E.P. (2019). BRD9 defines a SWI/SNF sub-complex and constitutes a specific vulnerability in malignant rhabdoid tumors. Nat. Commun..

[43] Sasaki M., Ogiwara H. (2024). Efficacy of glutathione inhibitor eprenetapopt against the vulnerability of glutathione metabolism in SMARCA4-, SMARCB1- and PBRM1-deficient cancer cells. Sci. Rep..

[44] Sasaki M., Kato D., Murakami K., Yoshida H., Takase S., Otsubo T., Ogiwara H. (2024). Targeting dependency on a paralog pair of CBP/p300 against de-repression of KREMEN2 in SMARCB1-deficient cancers. Nat. Commun..

[45] Mao B., Wu W., Davidson G., Marhold J., Li M., Mechler B.M., Delius H., Hoppe D., Stannek P., Walter C. (2002). Kremen proteins are Dickkopf receptors that regulate Wnt/beta-catenin signalling. Nature.

[46] Sumia I., Pierani A., Causeret F. (2019). Kremen1-induced cell death is regulated by homo- and heterodimerization. Cell Death Discov..

[47] Amara C.S., Kami Reddy K.R., Yuntao Y., Chan Y.S., Piyarathna D.W.B., Dobrolecki L.E., Shih D.J.H., Shi Z., Xu J., Huang S. (2024). The IL6/JAK/STAT3 signaling axis is a therapeutic vulnerability in SMARCB1-deficient bladder cancer. Nat. Commun..

[48] Wendt M.K., Balanis N., Carlin C.R., Schiemann W.P. (2014). STAT3 and epithelial-mesenchymal transitions in carcinomas. Jakstat.

[49] Liu W.H., Chen M.T., Wang M.L., Lee Y.Y., Chiou G.Y., Chien C.S., Huang P.I., Chen Y.W., Huang M.C., Chiou S.H. (2015). Cisplatin-selected resistance is associated with increased motility and stem-like properties via activation of STAT3/Snail axis in atypical teratoid/rhabdoid tumor cells. Oncotarget.

[50] Murzabdillaeva A., Elzamly S., Brown R., Buryanek J., Jafri S., Rowe J. (2020). Prometastatic CXCR4 and Histone Methyltransferase EZH2 are Upregulated in SMARCB1/INI1-deficient and TP53-mutated Metastatic Poorly Differentiated Chordoma to the Liver. Am. J. Clin. Pathol..

[51] Liu H., Liu Y., Liu W., Zhang W., Xu J. (2015). EZH2-mediated loss of miR-622 determines CXCR4 activation in hepatocellular carcinoma. Nat. Commun..

[52] Chien Y.C., Chen J.N., Chen Y.H., Chou R.H., Lee H.C., Yu Y.L. (2020). Epigenetic Silencing of miR-9 Promotes Migration and Invasion by EZH2 in Glioblastoma Cells. Cancers.

[53] Marhelava K., Pilch Z., Bajor M., Graczyk-Jarzynka A., Zagozdzon R. (2019). Targeting Negative and Positive Immune Checkpoints with Monoclonal Antibodies in Therapy of Cancer. Cancers.

[54] Jiang Y., Chen M., Nie H., Yuan Y. (2019). PD-1 and PD-L1 in cancer immunotherapy: Clinical implications and future considerations. Hum. Vaccines Immunother..

[55] Liu P.C., Ssu C.T., Tsao Y.P., Liou T.L., Tsai C.Y., Chou C.T., Chen M.H., Leu C.M. (2020). Cytotoxic T lymphocyte-associated antigen-4-Ig (CTLA-4-Ig) suppresses Staphylococcus aureus-induced CD80, CD86, and pro-inflammatory cytokine expression in human B cells. Arthritis Res. Ther..

[56] Bolm L., Petruch N., Sivakumar S., Annels N.E., Frampton A.E. (2022). Gene of the month: T-cell immunoreceptor with immunoglobulin and ITIM domains (TIGIT). J. Clin. Pathol..

[57] Davis K.L., Fox E., Isikwei E., Reid J.M., Liu X., Minard C.G., Voss S., Berg S.L., Weigel B.J., Mackall C.L. (2022). A Phase I/II Trial of Nivolumab plus Ipilimumab in Children and Young Adults with Relapsed/Refractory Solid Tumors: A Children’s Oncology Group Study ADVL1412. Clin. Cancer Res..

[58] Forrest S.J., Yi J., Kline C., Cash T., Reddy A.T., Cote G.M., Merriam P., Czaplinski J., Bhushan K., DuBois S.G. (2021). Phase II study of nivolumab and ipilimumab in children and young adults with INI1-negative cancers. J. Clin. Oncol..

[59] Nutsch K., Banta K.L., Wu T.D., Tran C.W., Mittman S., Duong E., Nabet B.Y., Qu Y., Williams K., Muller S. (2024). TIGIT and PD-L1 co-blockade promotes clonal expansion of multipotent, non-exhausted antitumor T cells by facilitating co-stimulation. Nat. Cancer..

[60] (2022). A Phase 1/2 Study of Tiragolumab (NSC# 827799) and Atezolizumab (NSC# 783608) in Patients with Relapsed or Refractory SMARCB1 or SMARCA4 Deficient Tumors. https://www.dana-farber.org/clinical-trials/22-528.

[61] Kang N., Eccleston M., Clermont P.L., Latarani M., Male D.K., Wang Y., Crea F. (2020). EZH2 inhibition: A promising strategy to prevent cancer immune editing. Epigenomics.

[62] Yi J.S., Czaplinski J., Bhushan K., Forrest S.J., Shukla N., Mack S., Hong A., Strachan M., DuBois S.G., London W.B. (2024). TRLS-15. “TAZNI”: A phase I/II combination trial of tazemetostat with nivolumab and ipilimumab for children with INI1-negative or SMARCA4-deficient tumors (NCT05407441). Neuro-Oncology.

[63] Sterner R.C., Sterner R.M. (2021). CAR-T cell therapy: Current limitations and potential strategies. Blood Cancer J..

[64] Dabas P., Danda A. (2023). Revolutionizing cancer treatment: A comprehensive review of CAR-T cell therapy. Med. Oncol..

[65] ClinicalTrials.gov Interleukin-15 Armored Glypican 3-Specific Chimeric Antigen Receptor Expressed in Autologous T Cells for Solid Tumors. ClinicalTrials.gov Identifier: NCT05103631. NCT05103631.

[66] ClinicalTrials.gov Interleukin-15 and -21 Armored Glypican-3-Specific Chimeric Antigen Receptor Expressed in T Cells for Pediatric Solid Tumors. ClinicalTrials.gov Identifier: NCT04715191. NCT04715191.

[67] ClinicalTrials.gov Interleukin-15 Armored Glypican 3-Specific Chimeric Antigen Receptor Expressed in T Cells for Pediatric Solid Tumors. ClinicalTrials.gov Identifier: NCT04377932. NCT04377932.

[68] ClinicalTrials.gov Study to ONO-4538 in Patients with Rhabdoid Tumor. ClinicalTrials.gov Identifier: NCT06622941. NCT06622941.

[69] ClinicalTrials.gov Immunotherapy for Malignant Pediatric Brain Tumors Employing Adoptive Cellular Therapy (IMPACT). ClinicalTrials.gov Identifier: NCT06193759. NCT06193759.

[70] ClinicalTrials.gov Loc3CAR: Locoregional Delivery of B7-H3-CAR T Cells for Pediatric Patients With Primary CNS Tumors. ClinicalTrials.gov Identifier: NCT05835687. NCT05835687.

[71] ClinicalTrials.gov B7-H3-Specific Chimeric Antigen Receptor Autologous T-Cell Therapy for Pediatric Patients with Solid Tumors (3CAR). ClinicalTrials.gov Identifier: NCT04897321. NCT04897321.

[72] ClinicalTrials.gov Study of B7-H3-Specific CAR T Cell Locoregional Immunotherapy for Diffuse Intrinsic Pontine Glioma/Diffuse Midline Glioma and Recurrent or Refractory Pediatric Central Nervous System Tumors. ClinicalTrials.gov Identifier: NCT04185038. NCT04185038.

[73] ClinicalTrials.gov EGFR806 CAR T Cell Immunotherapy for Recurrent/Refractory Solid Tumors in Children and Young Adults. ClinicalTrials.gov Identifier: NCT03618381. NCT03618381.

[74] ClinicalTrials.gov B7H3 CAR T Cell Immunotherapy for Recurrent/Refractory Solid Tumors in Children and Young Adults. ClinicalTrials.gov Identifier: NCT04483778. NCT04483778.

[75] Esteller M., Dawson M.A., Kadoch C., Rassool F.V., Jones P.A., Baylin S.B. (2024). The Epigenetic Hallmarks of Cancer. Cancer Discov..

[76] Sadida H.Q., Abdulla A., Marzooqi S.A., Hashem S., Macha M.A., Akil A.S.A., Bhat A.A. (2024). Epigenetic modifications: Key players in cancer heterogeneity and drug resistance. Transl. Oncol..

[77] ClinicalTrials.gov EZH2 Inhibitor Tazemetostat in Pediatric Subjects with Relapsed or Refractory INI1-Negative Tumors or Synovial Sarcoma. ClinicalTrials.gov Identifier: NCT02601937. NCT02601937.

[78] Chi S.N., Bourdeaut F., Casanova M., Kilburn L.B., Hargrave D.R., McCowage G.B., Pinto N.R., Yang J., Chadha R., Kahali B. (2022). Update on phase 1 study of tazemetostat, an enhancer of zeste homolog 2 inhibitor, in pediatric patients with relapsed or refractory integrase interactor 1–negative tumors. J. Clin. Oncol..

[79] Ge Z., Da Y., Xue Z., Zhang K., Zhuang H., Peng M., Li Y., Li W., Simard A., Hao J. (2013). Vorinostat, a histone deacetylase inhibitor, suppresses dendritic cell function and ameliorates experimental autoimmune encephalomyelitis. Exp. Neurol..

[80] ClinicalTrials.gov Vorinostat and Temozolomide in Treating Young Patients with Relapsed or Refractory Primary Brain Tumors or Spinal Cord Tumors. ClinicalTrials.gov Identifier: NCT01076530. NCT01076530.

[81] Hummel T.R., Wagner L., Ahern C., Fouladi M., Reid J.M., McGovern R.M., Ames M.M., Gilbertson R.J., Horton T., Ingle A.M. (2013). A pediatric phase 1 trial of vorinostat and temozolomide in relapsed or refractory primary brain or spinal cord tumors: A Children’s Oncology Group phase 1 consortium study. Pediatr. Blood Cancer.

[82] Nemes K., Johann P.D., Tüchert S., Melchior P., Vokuhl C., Siebert R., Furtwängler R., Frühwald M.C. (2022). Current and Emerging Therapeutic Approaches for Extracranial Malignant Rhabdoid Tumors. Cancer Manag. Res..

[83] Du R., Huang C., Liu K., Li X., Dong Z. (2021). Targeting AURKA in Cancer: Molecular mechanisms and opportunities for Cancer therapy. Mol. Cancer.

[84] Upadhyaya S., Campagne O., Robinson G.W., Onar-Thomas A., Orr B., Billups C.A., Tatevossian R.G., Broniscer A., Kilburn L.B., Baxter P.A. (2020). Phase II study of alisertib as a single agent in recurrent or progressive atypical teratoid rhabdoid tumors. J. Clin. Oncol..

[85] Green A.L., Minard C.G., Liu X., Safgren S.L., Pinkney K., Harris L., Link G., DeSisto J., Voss S., Nelson M.D. (2025). Phase 1 trial of selinexor in pediatric recurrent/refractory solid and CNS tumors (ADVL1414): A Children’s Oncology Group Phase 1 Consortium Trial. Clin. Cancer Res..

[86] Geoerger B., Bourdeaut F., DuBois S.G., Fischer M., Geller J.I., Gottardo N.G., Marabelle A., Pearson A.D.J., Modak S., Cash T. (2017). A Phase I Study of the CDK4/6 Inhibitor Ribociclib (LEE011) in Pediatric Patients with Malignant Rhabdoid Tumors, Neuroblastoma, and Other Solid Tumors. Clin. Cancer Res..

[87] ClinicalTrials.gov Study of Efficacy and Safety of Ribociclib (LEE011) in Combination with Topotecan and Temozolomide (TOTEM) in Pediatric Patients With Relapsed or Refractory Neuroblastoma and Other Solid Tumors. ClinicalTrials.gov Identifier: NCT05429502. NCT05429502.

[88] ClinicalTrials.gov A Study of Selinexor in People with Wilms Tumors and Other Solid Tumors. ClinicalTrials.gov Identifier: NCT05985161. NCT05985161.

[89] ClinicalTrials.gov Study of Palbociclib Combined with Chemotherapy in Pediatric Patients with Recurrent/Refractory Solid Tumors. ClinicalTrials.gov Identifier: NCT03709680. NCT03709680.

[90] Cesur-Ergün B., Demir-Dora D. (2023). Gene therapy in cancer. J. Gene Med..

[91] Kim S.S., Moghe M., Rait A., Donaldson K., Harford J.B., Chang E.H. (2024). SMARCB1 Gene Therapy Using a Novel Tumor-Targeted Nanomedicine Enhances Anti-Cancer Efficacy in a Mouse Model of Atypical Teratoid Rhabdoid Tumors. Int. J. Nanomed..

[92] McDermott J., Sturtevant D., Kathad U., Varma S., Zhou J., Kulkarni A., Biyani N., Schimke C., Reinhold W.C., Elloumi F. (2022). Artificial intelligence platform, RADR^®^, aids in the discovery of DNA damaging agent for the ultra-rare cancer Atypical Teratoid Rhabdoid Tumors. Front. Drug Discov..

